# Canadian taTME expert collaboration (CaTaCO) position statement

**DOI:** 10.1007/s00464-020-07680-8

**Published:** 2020-06-05

**Authors:** Antonio Caycedo-Marulanda, Carl J. Brown, Sami A. Chadi, Shady Ashamalla, Lawrence Lee, Peter Stotland, Usmaan Hameed, George Melich, Grace Ma, Francois Letarte, Ahmer Karimuddin, Fayez Quereshy, Terry Phang, Manoj Raval, Elena Vikis, A. Sender Liberman, Alexandre Bouchard, Phillipe Bouchard, Sebastien Drolet

**Affiliations:** 1 Kingston General Hospital, 76 Stuart Street, Kingston, ON K7L 2V7 Canada; 2 Queen’s University, Kingston, Canada; 3 St. Paul’s Hospital, Vancouver, Canada; 4 University of British Columbia, Vancouver, Canada; 5 Toronto Western Hospital, Toronto, Canada; 6 University Health Network, Toronto, Canada; 7 Sunnybrook Health Sciences Centre, Toronto, Canada; 8 McGill University Health Centre, Montreal, Canada; 9 Health Sciences North, Sudbury, Canada; 10 Northern Ontario School of Medicine, Sudbury, Canada; 11 North York General Hospital, Toronto, Canada; 12 Royal Columbian Hospital/Eagle Ridge Hospital, New Westminster, Canada; 13 CHU de Quebec, Québec, Canada; 14 Universite de Laval, Québec, Canada

**Keywords:** Position statement, Expert collaboration, Transanal total mesorectal excision, Safety, Implementation

## Abstract

**Introduction:**

Transanal total mesorectal excision (taTME) is a novel approach to surgery for rectal cancer. The technique has gained significant popularity in the surgical community due to the promising ability to overcome technical difficulties related to the access of the distal pelvis. Recently, Norwegian surgeons issued a local moratorium related to potential issues with the safety of the procedure. Early adopters of taTME in Canada have recognized the need to create guidelines for its adoption and supervision. The objective of the statement is to provide expert opinion based on the best available evidence and authors’ experience.

**Methods:**

The procedure has been performed in Canada since 2014 at different institutions. In 2016, the first Canadian taTME congress was held in the city of Toronto, organized by two of the authors. In early 2019, a multicentric collaborative was established [The Canadian taTME expert Collaboration] which aimed at ensuring safe performance and adoption of taTME in Canada. Recently surgeons from 8 major Canadian rectal cancer centers met in the city of Toronto on December 7 of 2019, to discuss and develop a position statement. There in person, meeting was followed by 4 rounds of Delphi methodology.

**Results:**

The generated document focused on the need to ensure a unified approach among rectal cancer surgeons across the country considering its technical complexity and potential morbidity. The position statement addressed four domains: surgical setting, surgeons’ requirements, patient selection, and quality assurance.

**Conclusions:**

Authors agree transanal total mesorectal excision is technically demanding and has a significant risk for morbidity. As of now, there is uncertainty for some of the outcomes. We consider it is possible to safely adopt this operation and obtain adequate results, however for this purpose it is necessary to meet specific requirements in different domains.

The transanal total mesorectal excision (taTME) is a novel approach to surgery for rectal cancer first described by Sylla et al. a decade ago [1]. The technique has gained significant popularity in the surgical community due to the promising ability to overcome technical difficulties of accessing the distal pelvis. Some of the theoretical and recognized advantages of this novel procedure include direct visualization of distal margin, sphincter preservation, access to the difficult pelvis, fewer conversions to open surgery, and a decreased length of stay [2–5]. The belief is that taTME can produce better quality mesorectal specimens and therefore improved oncologic outcomes [6, 7]. As with any new technique, unseen complications have surfaced, some with potentially devastating consequences for patients (e.g., urethral injuries, CO_2_ air embolisms) [8, 9]. The learning curve associated with this procedure has proven to be particularly challenging to master [8–13].

Recently, Norwegian surgeons issued a moratorium on the technique after encountering a high rate of local recurrences shortly after surgery with multifocal patterns of presentation [14]. Thus, the safety of the procedure has been called into question. In contrast, Hol et al. have recently published reassuring long-term outcomes from 2 Dutch high volume centers [15]. A recent editorial by Atallah et al. discussed these discordant outcomes [16]. The authors emphasized on the importance of the results of the ongoing prospective trials which should shine light on the safety profile of the technique [17–19].

TaTME surgery has been performed in Canada since 2014 by early adopters of the technique at different institutions. In 2016, the first Canadian taTME congress was held in the city of Toronto, organized by two of the authors [EV, SA] under the auspice of the University of Toronto, which was followed by the development of The Canadian taTME Proctorship Network, based in Toronto. This was an early attempt to ensure safe implementation of the procedure in the country. The current climate of controversy [14, 20, 21] has led adopters of taTME in Canada to recognize the need to create guidelines and recommendations related to the procedure. In early 2019, we established a multicenter collaboration [*The Canadian taTME expert COllaboration* (CaTaCO)] aimed at ensuring safe performance and adoption of taTME in Canada. Surgeons from 8 major rectal cancer referral centers with a collective experience of over 700 TaTME procedures from across Canada (CaTaCO members) met in Toronto on December 7th of 2019, to discuss and develop this position statement.

The objective of the statement is to generate expert opinion on the adoption and monitoring of the transanal total mesorectal excision (taTME) procedure in Canada, based on the best available evidence and authors’ experience to ensure safe and appropriate implementation and oversight of this novel operation in Canadian institutions.

## Methodology

### Creation of the CaTaCO consensus group

The CaTaCO working group was put together based on knowledge prior to this publication of centers considered to be high volume in managing rectal cancer patients in Canada. Furthermore, these centers are well-known in the Canadian context to be performing taTME consistently, and are a cohesive group of subspecialty surgeons (colorectal surgery and surgical oncology) from across the country. Center-selection was ultimately also dependent on academic affiliation of institutions and a known involvement in research and data-audits. The discussion was limited to Canadian surgeons given the unique resource-based concepts of the single payer Canadian Healthcare system. Non-TaTME surgeons were not included; the authors believe that an in-depth knowledge of the procedure, from the inclusion criteria of patients, practical experience with the potential pitfalls and required knowledge and skills for problem-solving, were critical to effectively contribute to the discussion.

### Creation of task-list and statement strata

Prior to the CaTaCO consensus conference, a number of informal e-discussions were held among all authors. Initial components of the discussion were based on the perception of the need to establish consensus from expert guidance. No specific literature review was performed in identifying these areas of concern. Two or three authors were assigned to each task to provide expert knowledge and best evidence reviews on each subtopic. The corresponding author tabulated all points of discussion, stratifying by key themes. The initial strata were as follows:

- Requirements for adopting/learning TaTME
- Proctoring and supervision
- Independent performance
- Indications and patient selection
- Operational requirements
  1. Equipment
  2. Institution
  3. Surgical technique
- Documentation and audit of results

Following the in-person consensus conference on December 7th, 2019, after a number of rounds of discussion, the article was then structured into the following four areas, addressing each of the components that served as the foundation for the elements of the Delphi approach.

- Setting
- Surgeon
- Patient selection
- Quality assurance

### Delphi methodology

Following the in-person discussion, 4 rounds of discussion ensued to modify statements in an attempt to approach consensus; the number of rounds was defined a priori. A total of 19 surgeons from the 8 participating institutions contributed towards the creation of the document. Approval of each statement was accepted when 90% agreement or more was reached among the members. Significant discordances in opinion were addressed during the rounds of Delphi discussion; areas that lacked agreement were disclosed in the document. The response rate for all rounds was 100% with participation of all involved surgeons.

For the purposes of this paper, *rectal cancer surgeon* refers to surgeons that regularly care for patients with rectal cancer. *High volume institutions* refers to centers where the expertise and the logistics for comprehensive management of patients with rectal cancer are available. These definitions and this document were drafted in accordance with the recently published standards of rectal cancer care in Canada by The Canadian Partnership Against Cancer (CPAC) [22].

For the present work there was no requirement for an approval request from the ethics review board.

## Scope

This document focuses on the need to:

- Ensure a unified approach among rectal cancer surgeons across the country with respect to the implementation of taTME as a novel procedure with significant technical complexity and potential for significant morbidity.
- Recommend baseline pre-requisites for the safe and patient-centered performance of taTME surgery in Canada.
- Provide a clear definition of the training and expertise required by surgeons and institutions to reach and maintain proficiency. In addition, it creates awareness of the importance of minimum volume to sustain proficiency.
- Optimize patient benefit through the avoidance of inappropriate patient selection.

This manuscript does not intend to regulate the performance of the procedure but to provide guidance, based on expert opinion, for surgeons and institutions considering either commencing or continuing a taTME program in Canada.

## Major considerations

### Setting

- The controversy around institutional volume of cases is not settled. However, there is evidence to indicate that concentrated skills and expertise play a pivotal role in short and long-term surgical and oncological outcomes in the management of patients with rectal cancer [23–25]. TaTME should only be performed at centers with expertise in “complex” rectal cancer surgery as per CPAC guidelines [22] [19/19—100%].
- In the Canadian environment, CPAC guidelines [22] define “complex” rectal cancer center requirements as those with expert physician care, medical support care for major complications of abdominal surgery, allied health care services, perioperative planning services, post-operative care services these centers have at least two subspecialty trained rectal cancer surgeons, plus experts in reconstructive pelvic surgery (urologist, gynecologist, orthopedic and plastic surgeons), and there must have expertise in transanal endoscopic surgery (TES) [19/19—100%].
- Our recommendation of having a *minimum institutional volume* of rectal cancer patients is based on the premise that volume enables surgeons to understand disease specific issues relevant to the operation; anatomical planes by way of pattern recognition and avoidance of either unnecessary procedural morbidity, and/or inappropriate patient selection [17/19—89.5%].
- We have recommended a minimal institutional volume of *25 extra peritoneal* rectal cancers per year (this number *is NOT* equivalent to the above mentioned minimum institutional volume of rectal cancer cases) [19/19—100%].
- As all rectal cancer patients should be presented at MDT discussion, those considered for taTME approach *must* be presented and discussed in the context of appropriateness for this surgical approach [19/19—100%].

### Surgeon

- The controversy around surgeon volume for rectal cancer surgery is not settled. CPAC guidelines do not state a minimum volume for competence. However, there is evidence to indicate that volume plays a pivotal role in short and long-term surgical and oncological outcomes in the management of patients with rectal cancer [26–28]. TaTME should be only performed by surgeons with adequate volume and proficiency in the technique. In addition surgeons should demonstrate expertise and proficiency with abdomino-pelvic minimally invasive surgery as well as TES [19/19—100%].
- Significant and appropriate training and expertise in taTME surgery should have been completed. We consider that ideally training should be integrated in a formal postgraduate education program at a high volume taTME center. Current acceptable alternate pathways include a cadaveric in-person training program, usually offered to surgeons with the requisite minimum number of rectal cancer and transanal procedures performed per year. This would then be paired with a proctorship network for in-practice surgeons, supervised by surgeons who satisfy eligibility criteria for proctorship [19/19—100%].
- Our group recommends that a proctor should be a surgeon who has a track record of volume and quality in taTME surgery. A minimum number of taTME procedures is expected to qualify as a proctor, since it is required to have the ability to deal with a large variation of taTME scenarios and unexpected complications. Proctors should have a minimum number of 50 cases as primary operator performed, as an indication that the learning curve for the procedure has been overcome [11] [17/19—100%].

### Patient selection

- Given the potential morbidity of the procedure, taTME should be reserved for patients in whom a significant benefit could be envisioned by the operating team. This could include optimizing the oncologic distal dissection, sphincter preservation or any of the other mentioned benefits above [19/19—100%].
- Our collaborative would not recommend the taTME approach for patients whose tumor is above the peritoneal reflection and body habitus is deemed favorable for conventional laparoscopic surgery, as the added operative time, potential surgical morbidity, resource needs among others could add to the patient’s potential negative outcomes [19/19—100%].
- We would recommend that surgeons exercise caution in the selection and possible avoidance of patients with threatened CRM. Furthermore, we feel strongly that T4 tumors should not be dissected transanally but visualization of the distal margin with sphincter preservation may be achieved using a transanal platform in such cases [18/19—4.8%].
- TaTME is indicated for tumors located in the mid and low rectum, difficult to access and dissect with a conventional laparoscopic approach. Examples of these situations would include [19/19—100%]
  - Extra-peritoneal rectal cancer in male patients.
  - Obese male patients.
  - Bulky tumors in a narrow pelvis.
- When consenting patients, we recommend disclosure of:
  - Experience
  - Uncertainty of oncologic and functional outcomes.
  - Risk of injury to pelvic nerves/vessels/male urethra.
  - Theoretical benefits [18/19—97.8%].
- Complex cases, defined above, are recommended to be referred to high volume taTME surgeons for consideration/discussion. These should not be attempted early in a surgeon’s taTME experience nor should these procedures be performed by low volume taTME surgeons. Additional difficult cases include reoperative pelvic surgery (previous rectal surgery, previous prostatectomy) and intersphincteric abdominoperineal resections (APR) [19/19—100%].
- Our collaborative would recommend extreme caution if performing non-intersphincteric APRs with the taTME approach. This was strongly recommended against by most of the authors but not all. No consensus was reached but the majority considered there is a lack of benefit when weighed against the risks and difficulties with pneumopelvic dissection [16/19—100%].

### Quality assurance (database/monitoring)

As a novel and disruptive surgical intervention:

- Given the recently highlighted, single jurisdictional concerns that have come up, all patients undergoing taTME should have their data entered into a registry or shared database [19/19—100%].
- Additionally, given the importance of internal audits, surgeons should strongly consider sharing their data as part of a regional or national cohort to help with the monitoring and enhancement of the procedure and technique specific outcomes [19/19—100%].
- We encourage Canadian rectal cancer surgeons performing taTME to participate in and join CaTaCO [19/19—100%].
- Severe injury/complications should be discussed and reviewed with taTME experts and proctors (urethral injury, multifocal (local) recurrence) to identify potential intraoperative concerns that can be identified and remedied [19/19—100%].
- We support performing a quality review at an institutional level of taTME outcomes vs. existing open/laparoscopic/robotic oncologic outcomes to ensure the procedure continues to provide patient-centered benefits relative to current standards [19/19—100%].

## Discussion

The “bottom up” technique has gained popularity at a global scale, more recently, controversy regarding safety aspects of the approach has arisen, mainly related to the process of implementation and the oncological outcome-profile. There was overall agreement among the authors about the need to generate consensus and determine practical implementation guidelines within the Canadian context.

We developed a framework based on different aspects of the process, focused on the performance of the procedure on patients with a cancer diagnosis. Framework aspects include: the setting; the surgeon; the patient; and results audits. There are existing publications [29, 30] addressing similar concerns. In those, the authors have included surgeons from different countries with different levels of expertise, they also sought input from non-taTME surgeons and other specialties (oncologists, radiologist, pathologists). We understand the management of rectal cancer is multidisciplinary, and this concept is integral part of our recommendations throughout, in addition we have incorporated our recently published rectal cancer CPAC guidelines [22], which clearly delineate the multispecialty approach of rectal cancer in Canada. Nevertheless for the purpose of the present document, we focused on the input and concerns from established taTME Canadian surgeons with an academic affiliation.

We encountered several commonalities with other publications, for instance the difficulty to define a specific caseload for both, surgeon and institution. We have made emphasis on the importance of volume and expertise, these have been linked to better outcomes in patients with rectal cancer in general [25, 27, 28, 31]. The most contentious point of discussion was the use of this approach for the performance of abdominoperineal resections, most participants opposed to recommend it, however, three of nineteen surgeons felt it is appropriate to use it for this indication. Caution was recommended, the risk of injury of the urethra seems to be higher in these type of cases [32].

It is a well-recognized that taTME is technically demanding and reaching proficiency is difficult [9], the number required to overcome the learning curve has been estimated to be between 40 and 50 cases [11, 33, 34]. As part of a Canadian effort to audit taTME results in the country we are currently conducting an assessment of the short and long-term oncologic outcomes of the CaTaCO centers.

Proctoring and mentoring are essential components to successfully master the technique, recommendations for these aspects have been made above. The ideal method from our perspective is integrated training as part of a postgraduate educational program, however, alternative pathways are available and have been implemented in Canada and other countries. A good illustration of the latter has been published by Australian surgeons [13].

In conclusion we propose a practical framework for the safe implementation of taTME. The structure of the framework is based on four major aspects that can be revised by surgeons and institutions in any order, with the goal to safely introduce this complex approach into practice, additionally the statement can used to assess the status of centers where performance of the procedure is already ongoing.
